# Covidseeker: A Geospatial Temporal Surveillance Tool

**DOI:** 10.3390/ijerph19031410

**Published:** 2022-01-27

**Authors:** Yulin Hswen, Elad Yom-Tov, Vaidhy Murti, Nicholas Narsing, Siona Prasad, George W. Rutherford, Kirsten Bibbins-Domingo

**Affiliations:** 1 Department of Epidemiology and Biostatistics, University of California San Francisco, San Francisco, CA 94158, USA; George.Rutherford@ucsf.edu (G.W.R.); kirsten.bibbins-domingo@ucsf.edu (K.B.-D.); 2 Bakar Computational Health Sciences Institute, University of California San Francisco, San Francisco, CA 94143, USA; 3 Research and Development, Microsoft Research, Herzliya 4672415, Israel; eladyt@microsoft.com; 4 Manki Business, Morganville, NJ 07751, USA; vaidhy@mankibusiness.com (V.M.); nicholas@mankibusiness.com (N.N.); 5 Department of Computer Science, Harvard College, Cambridge, MA 02138, USA; sionaprasad@college.harvard.edu

**Keywords:** geospatial tracking, digital applications, contact tracing, human mobility, public surveillance, COVID-19, coronavirus

## Abstract

Introduction: Geospatial temporal data derived from smartphones traditionally used for purposes of navigation may offer valuable information for public health surveillance and locational hot spotting. Our objective was to develop a web-based application, called Covidseeker, that captures continuous fine-grained geospatial temporal data from smartphones and leverages these data to study transmission patterns of COVID-19. Methods: This report describes the development of Covidseeker and the process by which it utilizes geospatial temporal data from smartphones and processes it into a usable format to study geospatial temporal patterns of COVID-19. We provide an overview of the design process, the principles, the software architecture, and the dashboard of the Covidseeker application and consider key challenges and strategic uses of capturing geospatial temporal data and the potential for future applications in outbreak surveillance. Results: A resource such as Covidseeker can support situational awareness by providing information about the location and timing of transmission of diseases such as COVID-19. Geospatial temporal data housed in smartphones hold tremendous potential to capture more depth about where and when transmission occurs and the patterns of human mobility that lead to increases in risk of COVID-19. Conclusion: An enormous and highly rich source of geospatial temporal information about human mobility can be used to provide highly localized discrete information that is difficult to capture by traditional sources. The architecture of Covidseeker can be applied to help track COVID-19 and should be integrated with traditional disease surveillance practices.

## 1. Introduction

Understanding human mobility is one of the first steps to preventing the spread of infectious diseases transmitted from person to person such as COVID-19. Mathematical models using human mobility data have been used to study disease dynamics such as SARS and novel influenza A/H1N1 viruses [1]. Models using human mobility data offer the potential to identify hot spots of transmission and likely locations of exposure to the pathogen [1]. Understanding human mobility enables us to identify the hierarchy and timing of the progression, as well as the social and environmental contextual risks of infectious disease pandemics such as COVID-19 [1]. Capturing these human patterns of mobility can help us understand how to develop more effective and timely strategies to prevent the spread of COVID-19.

Traditionally, mobility data collection has been conducted through travel logs and surveys, which may not be sufficiently granular or accurate to achieve the goals required during a pandemic. For instance, travel diaries based on self-report are often used to track mobility [2] but may not be as accurate due to recall bias. Applications that do collect geographic data are often sparse because data are only collected when the user is using the application and thus is inconsistent and does not provide continuous mobility collection. Many other COVID-19 applications do not collect geospatial temporal data. Covidseeker is an application that overcomes many of these limitations, including the ability to collect retrospective geospatial temporal data. Many other COVID-19 applications do not collect individual-level geospatial temporal data. Covidseeker overcomes the limitations of just analyzing human mobility data at aggregated zip code level but uses individual-level data to be able to understand mechanistic patterns that can identify high-risk human mobility patterns and human behaviors.

Coupled with testing information, beyond individual contact tracing, individual-level geospatial temporal data can provide information on how people behave during their testing, quarantining, and social isolation periods. We can also begin to compare human mobility patterns between those who test positive vs. those who test negative, which can help develop further public health recommendations on public safety and social behaviors that are associated with transmission of COVID-19.

Furthermore, Covidseeker requires that users give full consent and an active donation. Therefore, participation is more ethical than the passive collection of data as recruitment is based on volunteered participation versus opting out of a data donation. The data will be aggregated and de-identified using random IDs, so no involved party will be able to connect the data back to one’s identity. Furthermore, the raw data will not be shared with any external third party. Public Health officials will act upon the conclusions drawn from analysis of the aggregated and de-identified data.

Beyond individual contact tracing, geospatial temporal data provide the important advantage of identifying patterns and hotspots of high transmission. The large volume of granular data allows us to understand movement patterns that repeatedly result in high case counts and specific locations associated with prevalent transmission. This knowledge can allow focused studies of these locations and a better design and greater enforcement of social distancing guidelines where they are needed.

Although previous research has explored mobility networks [3], much of these data had been aggregated and could only be investigated at the area level. Individual-level human mobility data at a refined and more granular level are required to better understand the transmission of COVID-19. In this report, we describe the design and features of the web-based application called Covidseeker that captures continuous fine-grained geospatial temporal data from smartphones and leverages these data to study transmission patterns of COVID-19.

## 2. Surveillance Applications for COVID-19

Unlike contact tracing and exposure notification applications, whose goal is to provide notification of being exposed to COVID-19, Covidseeker is built to collect geospatial temporal data to investigate transmission patterns of COVID-19. For instance, the (Google/Apple) Exposure Notification (GAEN) system [4] is a framework and protocol specification developed by Apple Inc. and Google to facilitate digital contact tracing. The GAEN system uses Bluetooth to identify if a user has been in proximity to someone who tested positive for COVID-19. The main purpose of this technology is to notify users about their potential exposure to COVID-19 and give them information on the necessary precautions such as isolation and testing. GAEN captures no personal information or geospatial temporal data from the user that can be used for purposes of research or analyses of mobility patterns of COVID-19. The identification of areas of exposure or locations that users traveled to when they were positive are not collected through this system.

Aggregated mobility datasets exist from companies such as Cuebiq and Safegraph. These mobility datasets come from smartphone users who have agreed to share their locations with apps such as weather apps [5,6]. For instance, approximately 15 million people in the United States allow apps to track their location regularly [6]. These mobility data are provided at the aggregated and anonymized level and are compiled by geographic area such as a county or state. For instance, the median distance moved per day by devices in a census track is given. Individual-level information is not available; therefore, individual patterns of behavior and locations of travel cannot be obtained through this data source. This type of aggregated mobility data has been traditionally used to help measure the optimal locations for operating a business or for location-based advertisement targeting. For instance, an advertisement might be shown about bathing suits for those who frequently visit a swimming pool. In the case of the COVID-19 pandemic, this type of data has been able to show if certain populations are sheltering in place and following social-distancing rules. For instance, Cuebiq data showed that the average mobility of those living in areas with low average household income is consistent with a lower likelihood of sheltering at home than persons living in areas with higher incomes. However, these aggregate data do not provide information at the individual level to identify different paths or mobility patterns [7]. Moreover, data collected from these companies are solely location-based in nature. Participants are not asked any specific questions about their social distancing behaviors, their COVID-19 status, or whether they have experienced any symptoms typical of COVID-19.

CovidTrace is another app that is used to study the symptoms of COVID-19 and track the virus’ local spread [8,9]. The app works by asking users to log in daily and fill out a questionnaire on their symptoms. The collected data are collected in large data sets with a research aim of better understanding symptoms. The reported symptoms are used to identify high-risk geographical areas, connections to preexisting conditions, exposure of healthcare workers, and the speed of spread. The survey-based platform allows the collection of varied information apart from location, yet also creates a larger time commitment and high sampling bias.

Reviews of existing digital applications reveal a focus on contact tracing rather than transmission patterns. Table 1 provides a summary of the surveillance applications for COVID-19. Furthermore, these existing methods show low overall reliability with unreliable counts of contacts and low time efficiency [10,11]. While many existing mobile applications have shown success in direct contact tracing, no existing mobile application meets Covidseeker’s goal of leveraging granular mobility data to pinpoint hotspots of high transmission. Many of the privacy concerns faced by existing applications that impeded widespread public acceptance were reviewed and addressed in Covidseeker [10,11].

## 3. Materials and Methods

Covidseeker was designed in March 2020 in response to the COVID-19 pandemic as a digital tool to collect and curate refined geospatial temporal data from individuals’ smartphones to better understand transmission patterns and to identify of points of exposure of COVID-19. The strategy was to deploy Covidseeker to uncover the likely locations and time points of where and when persons would be most at risk for contracting or spreading COVID-19. We also sought to understand mobility patterns of risk and changes in behavior in relation to testing, social distancing measures, and geographic area. Covidseeker overcomes the limitations of only being able to analyze human mobility data at aggregated zip code level but uses individual-level data to be able to understand mechanistic patterns that can identify high risk human mobility patterns and human behaviors that are influenced by COVID-19 testing. Coupled with testing information, beyond individual contact tracing, individual level geospatial temporal data can provide information on how people behave during their testing, quarantining, and social isolation periods. We can also begin to compare human mobility patterns between those who test positive vs. those who test negative, which can help develop further public health recommendations on public safety and social behaviors that are associated with transmission. The University of California, San Francisco’s institutional review board classified this study as public health surveillance and exempt from further review.

### 3.1. Source of Geospatial Temporal Data

Covidseeker utilizes a joint mobile-friendly and web-based platform that captures locational data from smartphone devices. Covidseeker captures data from Google Maps Locational History Timeline, which aggregates data points from individual smartphone devices. The Google Maps application uses GPS navigation systems, Wi-Fi, and cell tower connections to determine the time and location of a user’s smartphone device [12]. When the location history feature is enabled, Google Maps continuously collects geospatial temporal data from the user’s smartphone with an average interval of 4.5 min and has an accuracy up to 20 m [13,14]. The position is presented in the form of a pair of coordinates and a radius, and the estimate area for the tracked device is enclosed by a circle [13]. The data are collected, stored in the “cloud”, and synced with Google Maps and the Google account of the user. This Google Maps Locational History is archived in Google Timeline and can be downloaded by users using Google Takeout. Google Timeline and Takeout are services provided by Google [15]. Prior research has compared the accuracy of Google Maps Locational History and traditional GPS logs and has shown that Google Maps Locational History is accurate at capturing the movement, location, and the time spent in different microenvironments [16]. Because of the ubiquity of use of Google Maps and the consistency of incoming data that are obtained when location history is turned on, capturing data from Google’s Locational History Timeline provides a rich source of mobility data from smartphone users and allows them to submit a treasure trove of retroactive data, unlike most app-based solutions.

### 3.2. Questionnaire

**Figure 1:**
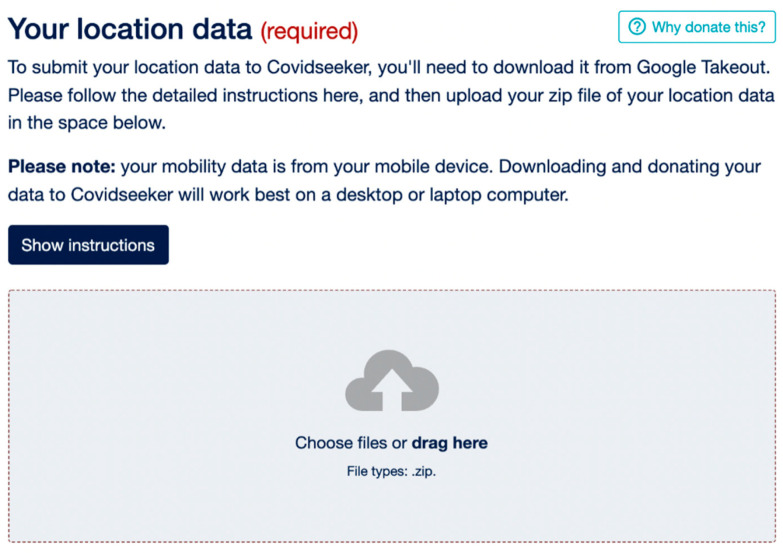
Covidseeker donation platform.

The Covidseeker platform asks participants to fill out a detailed survey, allowing the data to be analyzed in the specific context of COVID-19—submissions are seamlessly categorized into COVID-19 positive, negative, and not tested categories. On Covidseeker, users are first presented with a demographic and health survey. These questions consist of: Q1. What is your age? (in years); Q2. What is your gender? (Male, Female, Other, Prefer not to disclose) Q3. What is your home address? (Home address is used to calculate the percentage of time spent at home and the frequency and distance traveled from home. Home address is also excluded to de-identify the user’s information) Q4. Are you a healthcare worker? Q5. Have you experienced symptoms of COVID-19 such as cough or fever? If they answer yes to this question, users are asked what date they think they started having symptoms of COVID-19. Q6. Have you tested positive for an active infection with the novel coronavirus (the virus that causes COVID-19)? This is usually a nasal swab or saliva sample and is often referred to as a “PCR” test for the presence of viral RNA. If you have only tested positive based on antibodies without a test for active infectious, please answer no. Additional questions were included related to when they began experiencing symptoms of COVID-19 and if and when they had a positive test of COVID-19. We included a greater level of detail for this question because based on our user testing, users were unable to differentiate between a COVID-19 PCR test and an antibody test. Therefore, this question was expanded to clarify which test we were specifically inquiring about. If they answered yes to this question, users were asked the date they tested positive and where they think they contracted COVID-19 to help us better understand where a user might have been exposed. Q7. Have you been in contact with someone who tested positive for COVID-19? If they answered yes to this question, users are asked when were you in contact with someone who tested positive for COVID-19? This question was used to better identify changes in mobility if individuals know they have been exposed to someone with COVID-19. Q8. Is your smartphone an iPhone or an Android device? This question is asked for the purpose of identifying which operating system is more effective at tracking mobility. Users have the option of providing their email to be contacted in the future for more information about their mobility. A user enters the Covidseeker platform and enters their information through an online survey. After answering all the demographic questions, they are then prompted to download their geospatial temporal location data from their Smartphone device and upload it to the Covidseeker platform. Figure 1 shows the Covidseeker donation platform 1.

### 3.3. Location Data Importer

After the questionnaire, users are invited to donate their locational data from their smartphones. We provide information of why users should donate their data because they will help fight COVID-19 and establish ways to re-open our communities. Visual instructions on how to download their Google Maps Locational History from Google is provided to help users since a visualization familiarizes users with the process before they proceed. After they have downloaded their Google Locational History Timeline, users are able upload their location data to Covidseeker by choosing this file or dragging it to the box for uploading.

The purpose of the location data importer is to process the Google Timeline Location History exported from Google Takeout, a service intended to allow Google users to export their data from various Google services [14,17,18]. The importer only concerns itself with data from Google Location History, a component of Google Maps that uses smartphone GPS data to track users’ daily movements. Location History data are exported in two main types: semantic and raw. Semantic location history is organized by each distinct place the user visited, keyed by Google Place ID. Semantic locations include a name and address for each place. Raw location history is the set of latitude and longitude coordinates. Based on the user’s entry of home location to determine geocoordinates, Covidseeker determines the location is the user’s home by using a 100 m distance radius from the location given by the user and marks it as HOME if possible. Home coordinates are used to calculate hours spent at home or sheltering in place and distance traveled from home.

Covidseeker is programmed to only use one month’s worth of locational data that range from 2 weeks (14 days) before to 2 weeks after a user was tested for COVID-19. If the user was not tested for COVID-19, 30 days of data from the date of donation are kept. Users have the option of donating their entire locational history from their timeline for future studies. The choice to donate data is entirely voluntary.

### 3.4. Calculation of Timing and Location of Potential Exposures and Infection

The median time from exposure to symptom onset (incubation period) is estimated to be 5.1 days [19,20]. In other words, symptoms show in persons infected with COVID-19 approximately 5 days after contact. To identify the potential locations of exposure, Covidseeker uses 5 days before the onset of symptoms as the time when they were likely exposed to COVID-19. If the user is asymptomatic, Covidseeker takes 5 days before their positive COVID-19 test as their possible date of exposure. It is also estimated that the infectious time period begins 2 to 3 days before symptoms of COVID-19 present [21,22]. Thus, 3 days before the onset of symptoms is defined as the beginning of the infectious period, and 14 days after this date is considered the end of the infectious period. For asymptomatic users, 3 days prior to their positive COVID-19 test and 14 days after is considered their infectious period [23,24,25,26].

## 4. Results

### 4.1. Dashboard

**Figure 2:**
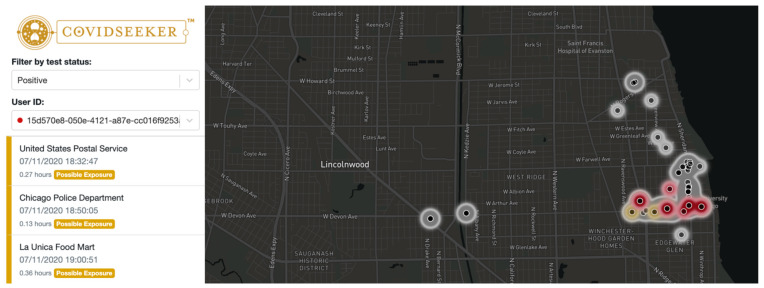
Dashboard of Covidseeker.

A dashboard was specifically created for the purposes of allowing researchers to see location data for each anonymized user. This is a password-protected page, and only the principal investigator of Covidseeker and those working on the project have administrative access; this access allows them to see location data for each user plotted on a geographic heatmap in order to help see where exposure is more likely to occur. Upon uploading location data to Covidseeker, geospatial temporal data are automatically processed and displayed visually with the amount of time spent in each location. Covidseeker collects both geocoordinates and semantic data, which are overlayed with Mapbox to identify exact locations names and the type of location. A Mapbox-based component that plots GeoJSON data from the application program interface (API) into different heatmap color layers: gold for locations where the user may have contracted COVID-19, red for places where the user was likely infectious, and grey for places where the user’s condition is unknown. Each layer is rendered as a Mapbox source layer. A larger cluster of points in an area results in a darker heat spot. For instance, latitude and longitude are collected and can be mapped onto the location if it is a restaurant or grocery store. The duration that someone spent at each location is also captured, enabling researchers to uncover if the length of time at a location poses a certain level of risk of COVID-19. The time periods and the subsequent locations of when the user was infectious are highlighted in red (Figure 2). Times and locations where users were likely exposed to COVID-19 are displayed in gold (Figure 2). All other time periods and locations are displayed in grey. The location name, type of location, and their time stamps are presented on the side dashboard as a list to help identify areas of transmission (Figure 2).

### 4.2. Data Security and Privacy

A random universally unique identifier (UUID) for the user ID is generated that is linked to the demographic and location data. The random UUID, survey data, and the geospatial temporal data are stored separately within their own separate database to increase privacy. For instance, we have a “primary database” server that contains user data and a “locations database” server that contains any location data imported for a user. This prevents the location data from directly identifying a user; the only identifier is a random UUID. The API never holds connections to both databases at the same time. Access is strictly limited to those with a direct connection to the project. The security of the data was approved by the University of California, San Francisco’s IT security.

## 5. Discussion

### 5.1. Key Challenges

Since Google Maps stores user location history with timestamps, retroactive geospatial temporal information can be captured longitudinally prior to the onset of disease. However, there are challenges to obtaining these retrospective data from Google Maps. The process is cumbersome, and those who are less technologically adroit may find it difficult, which could, in turn, lead to an attrition of users. We are unable to modify this process as developed by Google. Additionally, for location to be tracked continuously, location services have to be turned on to always in Google Maps. However, these retroactive geospatial temporal data are highly useful for studying transmission patterns of COVID-19 since they do not require users to download an additional application. Geospatial temporal data that are uploaded to Covidseeker are available for immediate analysis. Other smartphone applications collect geospatial temporal data prospectively, meaning that users would have to download the smartphone application before testing for COVID-19. It is less likely for users to have downloaded a smartphone application related to COVID-19 tracking prior to the pandemic or before the rise in the number of COVID-19 cases. To examine the transmission patterns of COVID-19, geospatial temporal data pre- and post-exposure to COVID-19 are necessary. Applications that collect prospective data would require user enrollment before testing, as retroactive data collection is not possible if the application was installed on the smartphone after the test date.

Competition for user’s time is a challenge. An abundance of COVID-19 applications have been released, and users may be overloaded with requests. This may be a barrier to the adoption of Covidseeker. In addition, there is a larger burden for those who tested positive for COVID-19, as many studies and applications are interested in obtaining their information, and users may feel too overwhelmed to participate. To offset these potential barriers, we have included information about how Covidseeker can provide more detailed and refined information about COVID-19 and how it is associated with an academic research institution.

Furthermore, there are limitations with the data captured from Covidseeker to be generalizable. Population-based sampling cannot be conducted, and thus, it is unlikely that the distribution of the data will reflect the United States population as a whole. Similar to other smartphone studies, persons that do not own a smartphone are excluded from Covidseeker’s data collection. Traditionally, individuals who do not own smartphones are lower-income, older, and from underrepresented minority populations, so they will not be as well represented in the results. This is important to note as little research exists on the mobility patterns of vulnerable population groups and the environmental risks they encounter in relation to COVID-19. Efforts will be in place to recruit minority and low-income populations to study their mobility patterns in order help reduce the burden of COVID-19 within these groups.

### 5.2. Advantages

Covidseeker offers the important advantage of collecting individual-level mobility information in order to study patterns of mobility that place a user at risk for COVID-19. By capturing refined geospatial temporal data, Covidseeker is able to identify the potential locations of exposure and behavioral changes at the individual level. For instance, Covidseeker has the potential to uncover the types of mobility patterns or locations that place persons more at risk for COVID-19. The data from Covidseeker can be also used to understand how patterns of mobility change based on a user’s test or their previous level of mobility or area of living. These questions have yet to be investigated. The collection of refined geospatial temporal patterns of mobility through Covidseeker could provide valuable information regarding transmission patterns of COVID-19 and future infectious diseases.

## 6. Conclusions

As public spaces such as restaurants, offices, stores, and schools begin to re-open, it is essential to have an effective methodology to predict risk, warn individuals about potential infection, and prevent the ongoing transmission of COVID-19 cases.

Using online anonymized aggregated privacy-safe geospatial temporal data, we can potentially identify locations conducive to high transmission and risky mobility patterns. Compared to traditional contact tracing methodologies, where single individuals are warned about potential infection and encouraged to self-isolate, Covidseeker focuses on identifying areas and patterns of high transmission in order to better inform large-scale safety measures.

While this Covidseeker tool focuses on the potential for data collection in the United States, the Covidseeker application demonstrates promise for applicability in other country contexts apart from the US. The utility of the app is limited by the willingness for users to donate their data—specifically, the active participation to Covidseeker vs. the passive retrieval of private information that is traditionally collected from big tech companies such as Google and Apple.

By identifying areas prone to high transmission, further investigation can be conducted into the specific practices that lead to increased risk such as failure to socially distance, high levels of contact, or lack of mask-wearing. Together, these data will provide insight into how to properly re-allocate resources to target high-risk practices and perhaps ease on restrictions in places that are not prone to high transmission. Answering these questions will allow us to determine when and where we can begin to reopen with an assurance of safety and informed preventive measures.

## Figures and Tables

**Table 1:** Comparison of existed digital technologies for understanding human mobility in response to COVID-19.

Specifications	Covidseeker	GAEN	Cuebiq	Safegraph	CovidTrace
Primary Use	Individual Mobility Patterns of Risk	Exposure Notification	Population Mobility Trends	Population Mobility Trends	Symptom Tracking
Data	Human Mobility	N/A	Human Mobility	Human Mobility	Survey Responses
Geographic Data	Individual	N/A	County Level	County Level	Individual
